# Roles of administration route, expectation, and belief in placebos in a randomized controlled trial with open-label placebos

**DOI:** 10.1038/s41598-025-27622-5

**Published:** 2025-12-02

**Authors:** Michael Schaefer, Carolin Liedtke, Sören Enge

**Affiliations:** Medical School Berlin, Rüdesheimer Str. 50, 14197 Berlin, Germany

**Keywords:** Open-label placebo, Placebo, Emotional distress, Embodiment, Expectation, Psychology, Human behaviour

## Abstract

Recent reports challenge the view that placebos need to be given in deception. Numerous studies report remarkable effects when giving placebos honestly in an open way. However, the mechanisms of open-label placebos still remain to be cleared. For example, while expectation is often regarded as an important mechanism for concealed placebos, it is unclear whether this process may also account for open-label placebo effects. Thus, alternative theories based on, for example, the embodiment theory are also discussed. Here we examined possible mechanisms of open-label placebos including administration route (to address rituals or sensorimotor embodiment mechanisms), expectation, and belief in placebos. Healthy participants viewed emotional distressing pictures and were given an open-label placebo beforehand or were in the control group. Results replicated previous studies by showing that participants in the open-label placebo conditions felt less emotional distress. Route of administration did not affect the placebo response. Treatment expectancy did not predict the placebo effect but was linked to the strength of the placebo effect that participants self-reported (which was not related to the actual placebo effect). Belief in open-label placebos was related to the strength of the placebo effect. Interestingly, an affirmative attitude towards homeopathic treatments was linked to a positive treatment expectation, but not to the actual placebo effect. We discuss the results with respect to different theories explaining open-label placebo mechanisms and argue that open-label placebos may work predominantly in an unconscious way.

**Supplementary Information:**

The online version contains supplementary material available at 10.1038/s41598-025-27622-5.

## Introduction

Until recently, it was taken for granted that placebos could only be administered covertly. Telling patients that they will receive placebos as a treatment would have been seen as ridiculous. However, recent studies suggest that beneficial effects can also be observed when informing the patients honestly that the pills they receive are placebos. Numerous studies report effects of an open-label placebo (OLP) treatment in patients with, for example, irritable bowel syndrome, chronic back pain, migraine, or cancer-related fatigue^1,2^. Moreover, OLP effects for healthy samples have also been shown, demonstrating, for example, reduced anxiety or emotional distress^3–5^.

How do open-label placebos work? While in conventional placebo studies expectation and classical conditioning are often regarded as main mechanisms for placebo responses^6–10^, their role for OLP effects is controversially discussed. Since the patients in OLP studies are told that there is no active ingredient inside the pills, expectation as the main mechanism may not seem convincing^11–13^. In many OLP studies patients are not only told that they will receive inert pills, but also honestly given the information that the experimenter does not know whether this treatment will help them^14^. Thus, in these studies neither the experimenter nor the pills promise or pretend that the treatment will work. Often, it is only be stated by the experimenter that OLPs *might* work (frequently with positive examples that OLPs have worked in other conditions). Therefore, when patients being asked about their expectation in OLP trials, they report low expectations and sometimes hope or openness to this new study design (“Let’s see what happens”,^15^). Consequently, a possible role for expectation seems much less convincing than in studies with concealed placebos.

However, previous findings on the role of expectation in OLP paradigms reported mixed results. On the one hand, several studies found that placebos with a plausible rational are more effective than without a rationale (e.g.,^16^). Furthermore, a recent network meta-analysis highlighted the importance of positive treatment expectations^17^. On the other hand, several studies did not find that positive baseline treatment expectations raised the OLP effect or were necessary to see an OLP effect at all (e.g.,^18^).

Similar to expectation, classical conditioning is seen as an important mechanism for placebos with deception, but in the special situation of OLP studies the role of this mechanism is not clear. On the one hand, it has been argued that often patients in OLP studies have a frequent history of experienced medical failures, making conditioning processes unlikely^19^. On the other hand, it has been shown that conditioned placebo analgesia persists even when patients know they are receiving a placebo^20^. However, OLP study designs do not include any formal conditioning processes^19^, but when OLPs are given within dose extension paradigms (conditioned OLPs), recent studies revealed encouraging results^21^.

Which mechanism might then explain OLP effects? Several alternative theories are discussed^22^. One approach argues that OLP effects might be understood with the embodiment theory^19,23,24^. This idea emphasizes two arguments. First, according to the embodiment approach, OLPs may work in an unconscious way. In this view, raising conscious expectations of positive treatment effects in OLP trials is not necessary. Consequently, there is no need to provide the usual extensive information that placebos have been shown to be powerful etc. In fact, some studies report that it is sufficient to provide patients just with an envelope saying “placebo”, without any explanation^25,26^. Second, the embodiment theory stresses the role of the body and its interaction with the environment. Embodied cognition is often explained by the example that we hear the whirling of a drill while sitting in the dentist’s waiting room. Soon this sound will make our mind start to imagine the drill, and our teeth may feel painful.

It is important to stress that this view is different from classical conditioning, because there is no necessary pairing before (maybe the dentist never used the drill for us). In the view of the classical conditioning theory, swallowing the OLP pill is the conditioned stimulus that will result in symptom relief due to the learned association between taking pills and symptom relief. However, previous work has demonstrated that OLP effects can be shown without actually swallowing the placebo pill^5^, which may suggest to focus more on other possible mechanisms^27^, because the effects take place without a possibly previously paired (physical) stimulus (although even imagining something might act as a conditioning stimulus^28^).

The embodiment theory argues that cognitive processes are based on an interplay between the mind, the body, and the world around us. Hence, in this view cognitive processes “are deeply rooted in the body’s interactions with the world”^29^. Thus, the body is seen as an interface between the mind and the world around us.

What does this mean? Wilson explains this with the example of counting on one’s fingers. We can do this by performing big movements for each of the different fingers. However, we can also do this in a more subtle way, allowing only the owner of the fingers to differentiate the fingers, which may look like twitching of the fingers for an observer. Wilson argues that we then may also “push the activity inward still further, allowing only the priming of motor programs but no overt movement. If this kind of mental activity can be employed successfully to assist a task such as counting, a new vista of cognitive strategies opens up”^29^. In this way, sensorimotor functions may subserve cognitive activities. Hence, sensorimotor simulations (e.g., the ritual of taking an inert pill twice a day for three weeks) may (unconsciously) affect our cognitions (as bottom-up processes) that in turn then reduce, for example, the perception of pain (via top-down processes). Previous studies have shown that the sensorimotor cortex is part of the pain matrix and has dense (back)projections to the thalamus and the insula, which are known as important structures in placebo analgesia^30,31^.

The present study aimed to address possible mechanisms of OLPs. We aimed to examine the roles of the administration route (as an attempt to use the ritual of taking a medicine to manipulate possible embodiment effects), positive treatment expectations, and belief in (open-label) placebo treatments. We used an established paradigm that has been shown to reliably produce OLP effects^32^. Healthy participants viewed emotional distressing pictures and received either an OLP nasal spray before or a nasal spray not labelled as a placebo. We manipulated the route of administration of the spray to address embodiment effects. Participants used the spray either on their own (a common medicine-taking ritual, high role of placebo-related sensorimotor engagement) or the experimenter provided the nasal spray to the subjects (less common medicine taking ritual, no placebo related sensorimotor engagement) (at least in adults nasal spray is usually self-administered). Furthermore, we examined the role of general positive treatment expectations at the start of the experiment, the belief in OLP effects and, the belief in placebos in general.

Having a nasal spray administered by another person can also involve important healthcare rituals, but as an adult, a nasal spray is usually taken by oneself (similar to pills but in contrast to vaccines). Nasal spray is usually given by someone else in the case of children (mean age of our participants was 26 years). Although memories of having a nasal spray administered by, for example, the parents may still be important, we think that the ritual of taking a nasal spray by oneself is stronger (or at least different) than the ritual (or the memory) of having administered a nasal spray by someone else.

Does the manipulation of the administration way reflect a manipulation of a healthcare ritual or a manipulation of embodiment? What is the difference between rituals and embodiment? Rituals can be described as structured sequences of actions (often with symbolic and social meanings). Embodiment is a theoretical concept that views the body as an interface between mind and environment. According to this view, we experience the world by and in the body and only later translate it into conscious meaning^33,34^. Thus, embodiment refers to the idea that cognition is grounded in bodily experience and action^35^. Rituals engage such embodied processes. For example, the physical act of taking a pill from a blister is not just a mechanical movement but also part of an embodied and symbolic practice associated with healing and health. Thus, rituals are a way in which embodied cognitions are enacted and reinforced^36^. In this view, the present study design attempts to investigate the role of embodiment by manipulating healthcare rituals.

Taking a pill for beneficial reasons with the knowledge that there is nothing inside is not only the description of OLP treatments. Perhaps relatively similar treatments are homeopathic medicaments that often do not have any effective pharmacological treatments inside at all, which is known by the patients. Do individuals who believe in the power of homeopathic treatments profit more than other participants from an OLP treatment? The current trial also aims to exploratory examine whether the belief in homeopathic treatments is linked to OLP effects.

## Materials and methods

### Participants

**Fig. 1:**
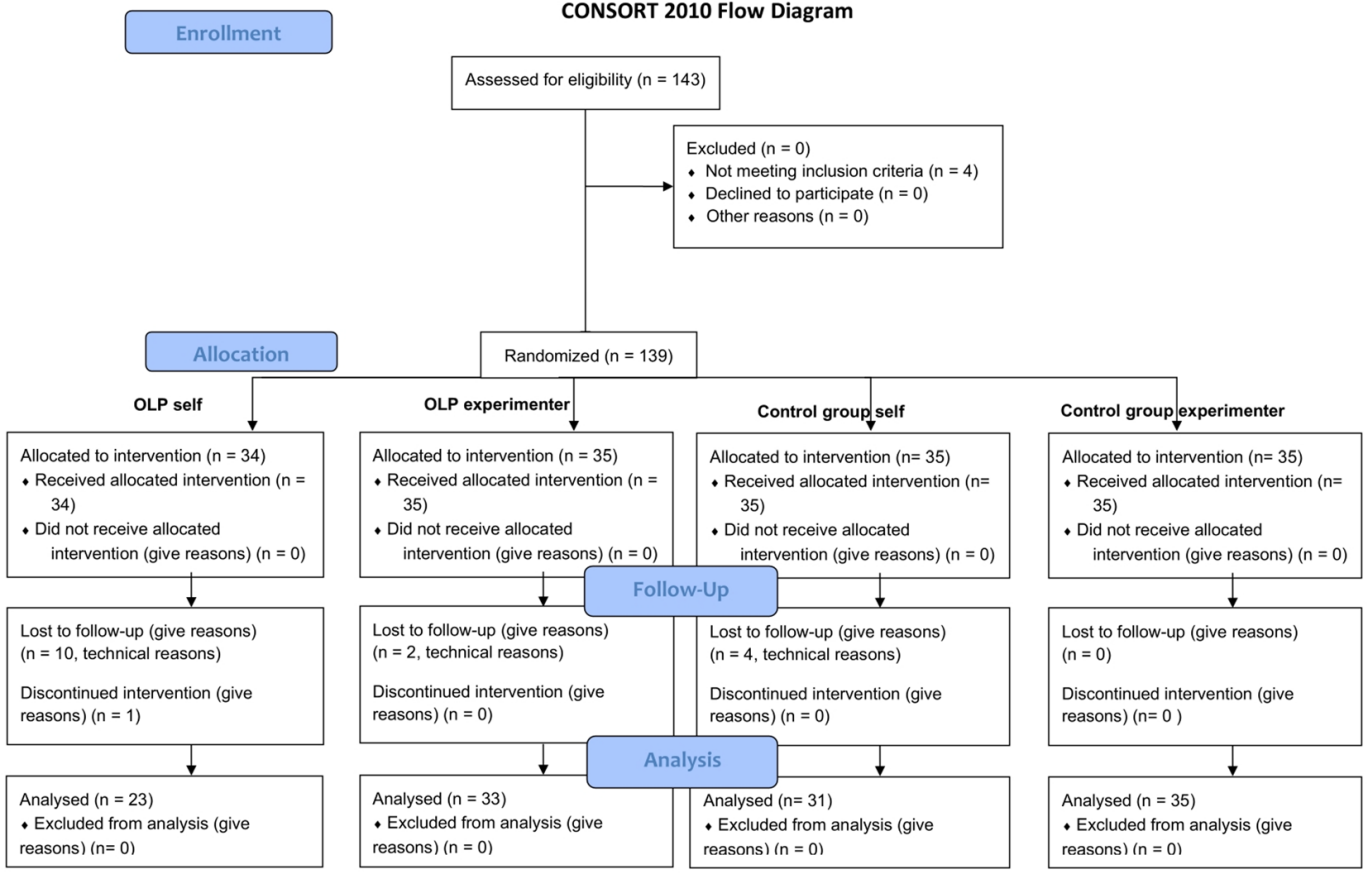
Flow diagram.

One hundred and twenty-two healthy participants with no neurological or psychiatric history took part in this study (mean age 26.28, standard deviation ± 5.64 years, 79 females, see FlowChart, Fig. 1). Participants were recruited via flyers at local universities. All participants gave written informed consent. The study adhered to the Declaration of Helsinki and was approved by the ethics committee of the Medical School Berlin.

Inclusion criteria was age between 18 and 50. Exclusion criteria were any known psychiatric or neurological history and pregnancy. The study was pregistered at the German Clinical Trail Registration (DRKS00035720, registered at 16/12/2024).

### Study design

The study consisted of a full-factorial design with two factors. The first factor (placebo) was receiving an OLP nasal spray with NaCL labelled as “Placebo” vs. receiving an identical nasal spray without placebo label. The second factor (manipulation of administration to address the ritual and sensorimotor engagement) was receiving the nasal spray administered by the experimenter or by the participant her- or himself. Thus, participants were randomized to one of four conditions. Participants first viewed pictures that were emotionally distressing or neutral, then they received the nasal spray and viewed again pictures that were emotionally distressing or neutral. In addition, we asked subjects about their beliefs in placebos or OLPs and their general expectation.

Sample size was determined based on previous OLP research on emotional distress^37^. A desired power of 0.95, a conservative effect size of f = 0.22, and an alpha error probability of 0.05 resulted in a required sample size of 112 participants, according to GPower^38^. To account for possible dropouts, we aimed to enroll a total sample size of about 140 participants.

### Procedure

The study was introduced to the participants as an experiment on the perception of emotional pictures. Participants first completed questionnaires about their trait anxiety (STAI-T) and their current mood (PANAS). The STAI-T is the German version of the State-Trait-Anxiety-Inventory (trait version)^39,40^. It s widely used to assess general anxiety feelings. The PANAS is the Positive and Negative Affect Schedule that includes 10 items of positive and 10 items of negative affective states^41^.

Then all participants were shown emotional and neutral pictures analogue to previous studies^32,37^. Thus, participants viewed 30 negative and 10 neutral pictures in a randomized order (identical to^37^). Pictures were shown for 6000 ms, then a fixation cross showed up for 4000 ms, followed by a picture rating period for 5000 ms. Here participants were asked to rate how the image made them feel on a nine-point Likert-scale ranging from 1 (not at all negative) to 9 (very negative). Pictures were taken from the IAPS data base^42^.

After completing this first picture viewing task participants were shown one of two presentations. Participants in the OLP conditions read a presentation about the power of placebo effects and that studies found that even placebos without deception had effects. At the end of the presentation the experimenter showed the participant a nasal spray that was labelled „Placebo “ (including the logo of the university) and announced that the participant now would receive a placebo spray. Depending on the condition then either the participants used the nasal spray on their own (one application to each nostril) or the experimenter applied the spray to the nose of the subjects. The participants were told that they will be given a placebo nasal spray to reduce their “negative emotional reactions. Again, this is a placebo, which means it does not contain any active ingredients, only saline solution, and it is completely harmless. But as you have read from the presentation, if you believe that the nasal spray will reduce your negative emotional reactions, then it actually will.” (taken from^32^).

Participants in the control conditions read a presentation about neurological processes of pain and the treatment of pain (both presentations were taken from^32^; presentations of both groups were comparable with respect to length, valanced words etc., see^32^). When participants reached the end of the presentation, the experimenter applied the nasal spray in a similar way (either the participants on their own or the experimenter used the spray), but in contrast to the placebo conditions the experimenter here introduced the nasal spray as including just water, which was necessary to obtain better physiological readings for some measurements we would do later and explain after the next step (analogue to^32^). The nasal spray was labelled „Nasal spray “(with the logo of the university). The experimenter paid attention that participants of these control conditions were not aware that other individuals participated in the same study but received a placebo nasal spray.

All participants then started the second part of the image viewing task, which was analogue to the first one (images were not identical to the first run). After completing this second image viewing task, we examined the belief in placebos in general by applying 4 items that have been used in previous studies (taken from^43^, see Supplementary Material S1) and the belief in OLPs, based on items taken from^32^ (5 questions that were embedded in other more general questions) (Supplementary Material S2). Moreover, we asked the participants to assess how much they believed that the nasal spray may have reduced the emotional distress during the picture viewing task (visual-analogue-scale (VAS), placebo conditions only). Similarly, we asked the participants (placebo conditions only) at the beginning of the study how much they expect that the placebo treatment will reduce their negative feelings in the current trial (VAS, Supplementary Material S3). Finally, we also asked the participants about their attitude with respect to homeopathic medical treatments using 4 items (Supplementary Material S4). At the end of the experiment all individuals were asked to rate the presentation (analogue to^32^, for control reasons) and participants of the control condition were debriefed about the true purpose of the nasal spray.

### Outcome measures

Our primary outcome measure was the change (pre-post) in emotional distress ratings (mean responses on the 9-point Likert scales for the negative pictures). There were no secondary outcome measures. However, we examined explorative outcomes. Thus, as outlined above, we examined the positive treatment expectation of the participants (VAS pre), the belief in OLPs and in placebos in general (after emotional distress ratings), and the rating whether the placebos had reduced the emotional distress (VAS post).

### Randomization and blinding

The group assignment was randomized by employing a computer-generated random number sequence and a sequentially numbered list. Obviously, participants in the OLP group knew about their group assignment. However, the control group was not aware that there is another group that receives the same treatment labelled as a placebo.

### Statistical analysis

Main outcome was the change from pre to post assessments of the emotional distress while viewing the pictures. To test our hypotheses, we calculated a two-factorial ANOVA using group (placebo vs. control) and administration route (self vs. experimenter; baseline data, age and STAI-T as covariates) as factors and change scores (pre to post) of emotional distress as dependent variable. Covariates were chosen to control for potential baseline differences as well as differences in general anxiety. Given the relatively wide age range of our sample, we also included age as a further covariate. To further evaluate whether expectation variables predict OLP effects we computed a linear regression analysis including positive treatment expectation, belief in placebo, and belief in OLP as predictors of emotional distress in the OLP groups (explorative analyses). For all statistical analysis the SPSS software package (IBM Corp., Armonk, NY, USA) was used and *p* values of < 0.05 were considered as significant.

## Results

Demographic details and baseline characteristics are shown in Table 1. OLP and control group data demonstrated that participants of the groups were comparable before starting the experiment. Results for mood (measured with PANAS) and ratings of emotional distress at baseline (first viewing of the images) were not different between the groups. In addition, groups were comparable with respect to trait anxiety (STAI-T, *p* > 0.10).

**Table 1:** Demographics and baseline characteristics (mean ± standard deviations).

Characteristic	Open-label placebo		Control group	
Administration	Self	Experimenter	Self	Experimenter
N	23	33	31	35
Age (in years)	25.52 ± 5.29	26.67 ± 5.25	25.52 ± 5.10	26.80 ± 6.73
Females / males	14 / 9	21 / 12	20 / 11	24 / 11
PANAS pos	2.42 ± 0.54	2.39 ± 0.80	2.46 ± 0.63	2.43 ± 0.77
PANAS neg	1.90 ± 0.50	1.84 ± 0.50	1.95 ± 0.50	1.90 ± 0.45
Baseline ratings neutral pictures	1.58 ± 0.33	1.78 ± 0.57	1.87 ± 0.56	1.88 ± 0.52
Baseline ratings negative pictures	6.09 ± 1.21	6.64 ± 1.42	6.85 ± 1.53	6.79 ± 1.25
Rating of presentation	6.50 ± 1.45	6.72 ± 1.12	6.14 ± 1.32	6.27 ± 1.60

**Fig. 2:**
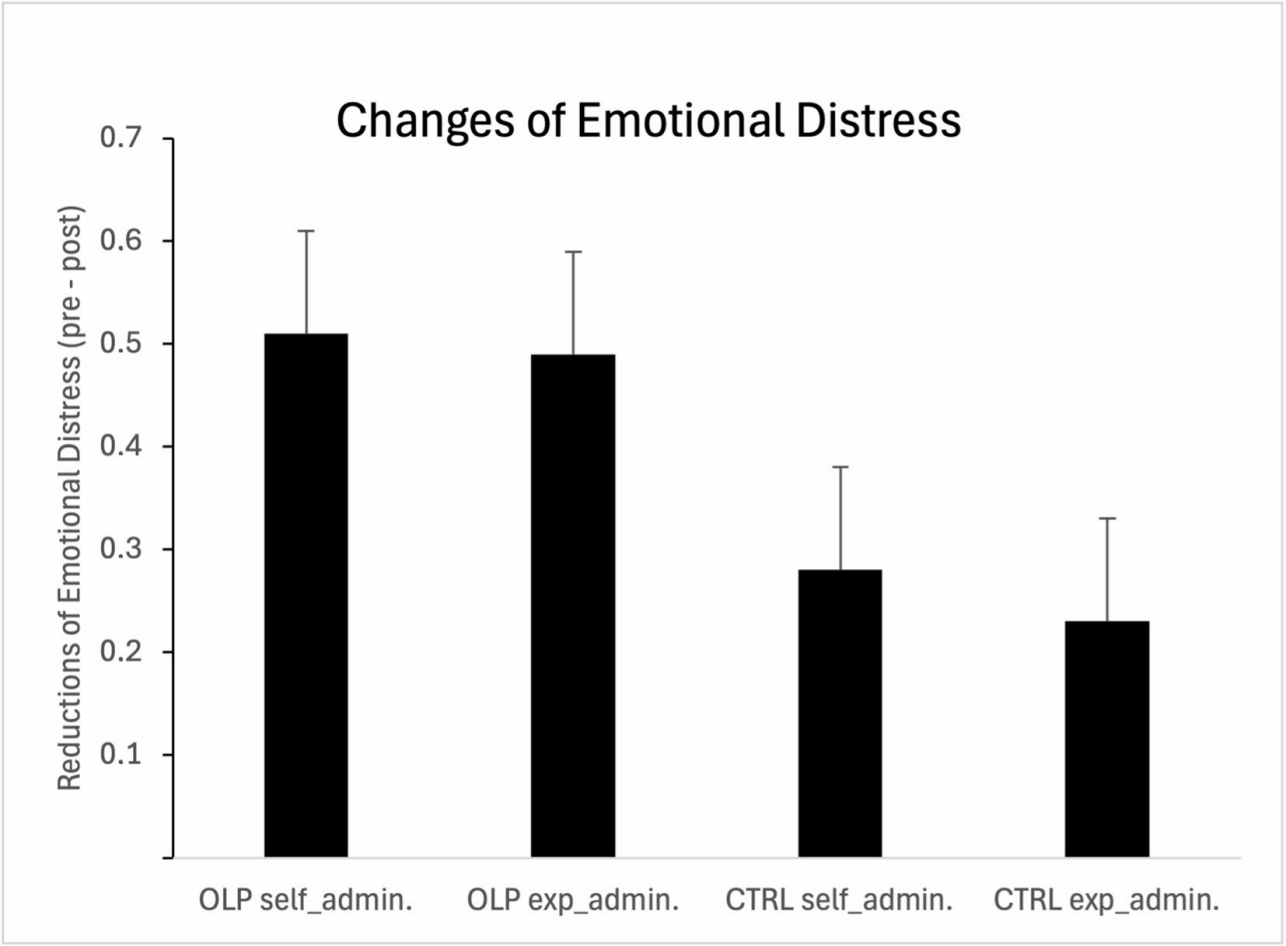
OLP effects on emotional distress (pre to post). Results demonstrate that OLP show higher reductions of emotional distress compared to the control group. Administration route (self vs. experimenter) did not affect the outcome parameter.

To test our hypotheses, we calculated change scores from pre to post of the ratings of distressing pictures. Results demonstrate that participants of the OLP group showed higher reductions after receiving the nasal spray compared with the control group, while route of administration did not affect the outcomes (Fig. 2). An ANCOVA with two factors (group and administration route; baseline data, age, and STAI-T as covariates) revealed a significant main effect for group (F(1, 115) = 4.30, *p* = 0.040, partial eta^2^ = 0.04), replicating the OLP effect on emotional distress (similar significant results when computing an ANCOVA including time (pre-post) as an additional factor (instead of change scores): interaction between time and placebo vs. control, F (1, 116) = 4.03, *p* = 0.047, partial eta^2^ = 0.03, no other significant results). We did not find a significant main effect or interaction for the factor administration route, suggesting that this variable did not affect distress ratings or interacted with the OLP effect (all *p's* > 0.10). An analogue calculation for neutral pictures revealed no significant main effects or interactions.

**Fig. 3:**
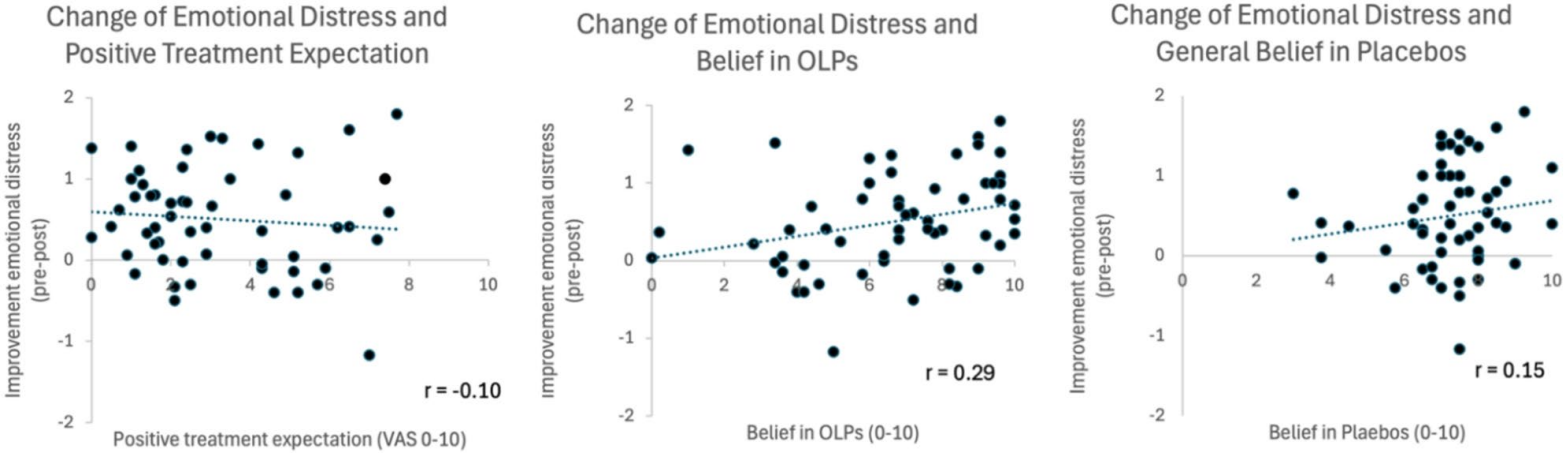
Scatterplots of relationship between changes in emotional distress, positive treatment expectations, belief in OLP and general belief in placebos (OLP group only). Only belief in OLPs demonstrated a significant relationship (Pearson correlation, r = 0.29, p = 0.032, two-sided).

We then examined whether expectation or belief in placebo or OLPs predicted the improvements in emotional distress from pre to post (change score, OLP group only). We calculated a linear regression model in which these predictors went simultaneously into the analysis (positive expectation of the treatment at the beginning of the trial, belief in placebos in general, belief in OLPs). The model failed to reach the level of significance, and no significant predictors were shown (R = 0.32, adjR^2^ = 0.05, F(3,54) = 1.94, *p* = 0.136). Adding age and gender as further predictors led to an improved regression model with a trend to significance (R = 0.42, adjR^2^ = 0.100, F(5,54) = 2.20, *p* = 0.070). The belief in OLPs was a significant predictor (β = 0.31, *p* = 0.032), expectations at the beginning of the trial or belief in placebos in general did not predict OLP effects (*p* > 0.10). However, a further significant predictor was age (β = 0.28, *p* = 0.042). Figure 3 depicted a scatterplot of these relationships.

Although the means of the placebo groups in the belief in OLPs were higher than in the control conditions (as expected due to the different instruction), this difference failed to reach the level of significance (*p* = 0.204) (see Table 2). However, it has to be stressed that the questions with respect to the belief in placebos was completed at the end of the experiment, whereas the presentation and information about placebos and OLPs was given at the beginning of the experiment (after first viewing pictures).

**Table 2:** Placebo beliefs (on a scale from 0 (very low) to 10 (very high)) and treatment expectation (VAS scores) (mean ± standard deviations). See text for further details.

Characteristic	Open-label placebo		Control group	
Administration	Self	Experimenter	Self	Experimenter
Positive treatment expectation	3.04 ± 2.51	3.32 ± 1.93	–	–
Belief in OLP	6.72 ± 2.27	6.58 ± 2.79	6.28 ± 2.21	6.02 ± 2.63
Belief in placebos in general	7.46 ± 1.51	7.14 ± 1.26	6.63 ± 1.49	6.95 ± 1.40

To further examine the relationship between the five questions addressing the belief in OLPs and the question about treatment expectation we exploratory computed a factor analysis of these variables. Results of a principal component analysis revealed two factors, which explained 80% of the total variance. All of the five questions of the belief questionnaire loaded high on the first factor (> 0.80), whereas only expectation loaded high on the second factor (0.98) (see Table 3). Thus, the question about the expectation seems to be different from the questions about the belief in OLPs.

**Table 3:** Results of factor analyses of questions about the belief in OLPs and expectation. See text for further details.

	Component 1	Component 2
Belief in OLPs: A placebo can still work on me even though I know that I am taking a placebo	0.93	0.02
Belief in OLPs: In order for placebos to work, the person needs to be deceived into believing they are taking an actual medicine	0.91	0.06
Belief in OLPs: A placebo can reduce my negative emotions even though I know I am taking a placebo	0.86	0.21
Belief in OLPs: A placebo only works if the person is deceived into thinking they are taking an actual medicine	0.84	−0.10
Belief in OLPs: A placebo can reduce my pain even though I know that I am taking a placebo	0.80	−0.07
Expectation: In this study, we are attempting to reduce your unpleasant feelings through a special placebo treatment. How likely do you think this placebo treatment is to reduce your negative feelings?	−0.12	0.98

At the end of the experiment, we asked the participants how much they think the OLP nasal spray may have reduced their emotional distress. It seems remarkable that the participant’s ratings were only weakly linked to the realistic reductions of their ratings during viewing the pictures (r = 0.17, two-sided, *p* = 0.212). Thus, the participants did not seem to be aware how much the OLP may have worked. This is also supported by a significant correlation between this perceived reduction (VAS post) and the positive treatment expectation (VAS pre) at the beginning of the experiment (r = 0.27, *p* = 0.044). Considering that the positive treatment expectation (VAS pre) was not linked to the actual reduction of distress (see above) it can be concluded that the prospective expectation and the retrospective assessment of success of the treatment are linked with each other, representing the participant’s attitude towards this novel treatment. However, these conscious thoughts about the treatment were not related to the actual impact of the OLP treatment.

**Fig. 4:**
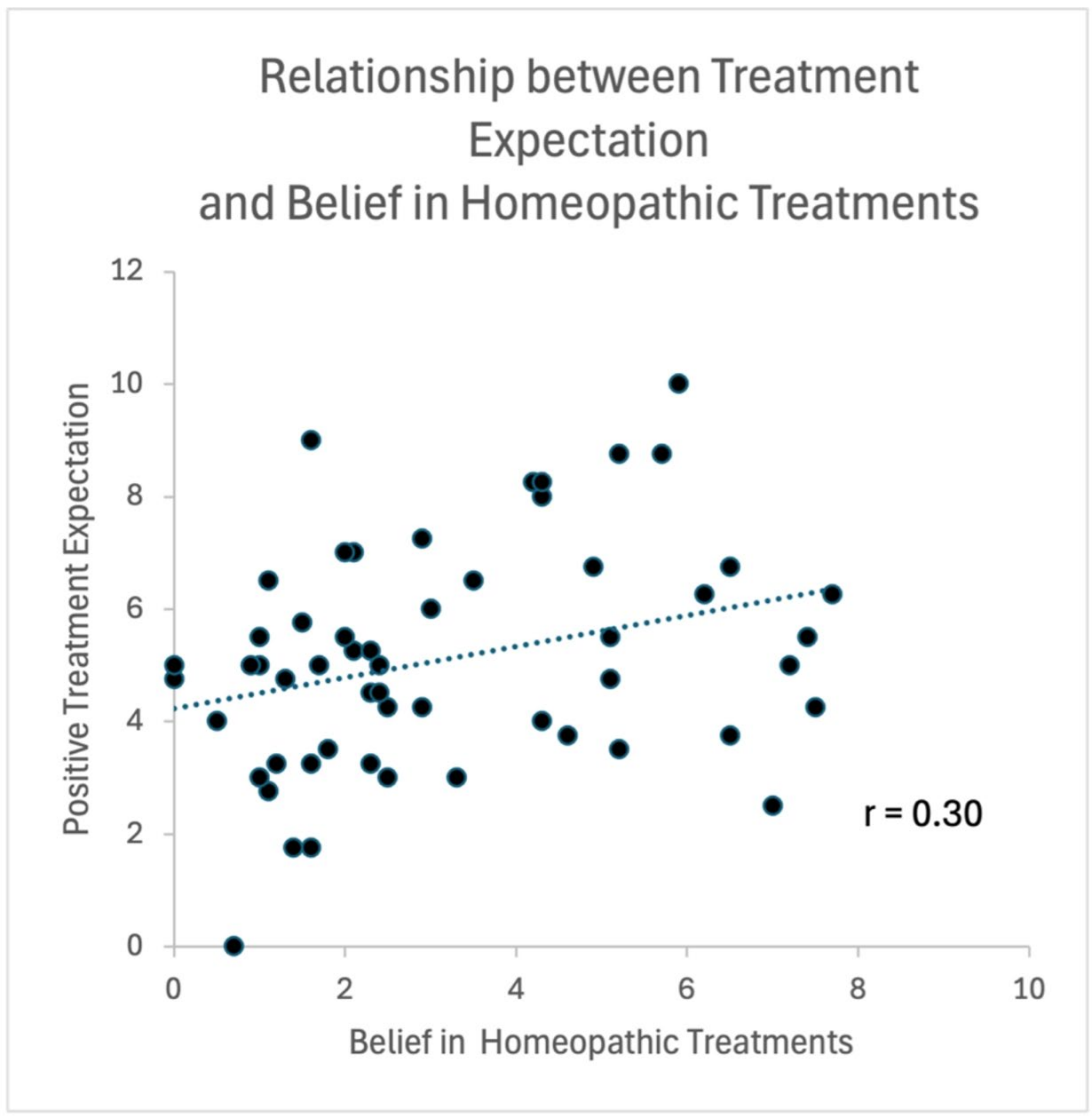
Scatterplot of correlation between belief in homeopathic treatments and positive treatment expectation for placebo treatment (OLP group only). See text for further details.

We did not find that the belief in homeopathic treatments was linked to the improvement in distress in the OLP condition or the belief in (open-label) placebos (all *p*’s > 0.10). However, we found that homeopathic belief was correlated with the positive treatment expectation (r = 0.30, *p* = 0.028, Fig. 4). Thus, the more participants tended to believe in the power of homeopathic medicaments, the more individuals also believed that the OLP treatment will work. Furthermore, results revealed that participants who believed in the power of homeopathic treatments also rated the negative pictures as more negative (r = 0.26, *p* = 0.003), suggesting that those individuals show higher general sensitivity to the distressing stimuli. Can this be explained by a higher level of general anxiety? This seems unlikely, since the results of the STAI were not linked to the homeopathic belief (r = 0.09, *p* > 0.10).

## Discussion

The current study aimed to examine the mechanisms by which OLPs work. We found that the administration route was not important for the OLP response, but (in contrast to the positive treatment expectation at the beginning) the belief in OLPs was positively linked to the placebo effect.

To test the role of different possible mechanisms we employed a paradigm that has been reported before to show OLP effects^32,37^. Our study may reflect an improved study design since we included baseline measurements. We replicated these previous results by revealing a similar effect of stress relief in OLP conditions. Thus, OLPs can relief emotional stress induced by images.

It is obvious that a placebo pill (or nasal spray) itself does not result in beneficial effects^44^ (neither in concealed nor in open placebos), since the pill has no active ingredients. Consequently, a recent study demonstrated placebo effects when participants only imagined taking an OLP pill, without actually swallowing it^5^. OLP appears to function as a result of a multimodal behavioral intervention (including cognitive, social, and physiological dimensions)^19^, but it remains unclear whether positive treatment expectations, embodiment processes, belief in OLP, or something else is crucial here.

A first mechanism this work aimed to address was the role of embodiment or the ritual of taking a medical treatment. According to the embodiment theory the body plays the role of an interface between mind and body (e.g.,^35,45,46^). Thus, sensorimotor facets (or sensorimotor memories) might contribute to the beneficial effects of an OLP treatment. In the current study we tried to test this role of sensorimotor contributions for OLPs by manipulating the role of administration. Therefore, we examined whether it makes a difference when taking the placebo on the own or when given by an experimenter. When the participants take the nasal spray by themselves, this includes handling the nasal spray, holding it, using it and putting it down again. These sensorimotor components may activate embodied motor memories of the ritual of taking a medicine in an unconscious way. Thus, we hypothesized that taking the placebo on the own would show a stronger OLP effect than just passively receiving the nasal spray.

However, our results revealed no effects of the administration route. One could speculate that a more frequent placebo intake would make a difference here (e.g., many studies provide placebo pills twice a day for three weeks). Another explanation might be that the situation was not adequately “private” to engage embodiment processes (to activate embodied sensory or motor memories); perhaps taking a placebo at home may have resulted in different outcomes. However, it seems difficult to experimentally manipulate embodiment variables. Here we tried to vary the sensorimotor contribution when taking a (single) placebo to activate sensorimotor memories of the medical ritual, but embodiment effects also take place when simply observing (and thereby being reminded on) a movement rather than moving the body itself^47,48^. Thus, OLP effects have been reported even when people merely imagined taking a pill^5^. Moreover, administering the nasal spray by the experimenter may also involve participant’s peripersonal space, which might affect the relationship between participants and experimenter^49^. In addition, having a nasal spray administered by someone else may have evoked memories of childhood for the participants (when their parents administered nasal spray to them), reminding them of an early healthcare ritual.

Thus, further attempts are necessary to unravel possible embodiment mechanisms in OLP paradigms. One could speculate that other kinds of sensorimotor experience than those manipulated in the current study might be important. For example, different sensorimotor experiences depending on the forms of application may affect the OLP effect. Winkler et al. investigated the role of the pharmaceutical form in an OLP study and found that OLP nasal spray is superior to OLP capsules^50^. Since these different application forms are associated with different sensorimotor contributions, the reason for this result might point to different embodied cognitions.

Although the manipulation of sensorimotor aspects engaged with the placebo intake did not reveal any effects, the embodiment theory still seems to be very promising to explain why OLPs may work. Several concepts in psychology draw our attention to similar effects in other research areas. For example, the illusory truth effect describes the tendency to believe false information when repeatedly presented. Remarkably, it has been reported that even knowledge does not protect against this effect^51^. For example, even if we later realize that a story we were told is actually not true, we sometimes still have a bad impression about the protagonist. In an evolutionary sense, somatic feelings developed much earlier than rational thinking. However, the latter did not replace the older one, rather it developed on the foundation of the previous one. Therefore, unconscious (for example, somatosensory) information can sometimes significantly influence our decisions and impressions^52–54^. OLPs with its somatosensory dimensions may be another example of these fast unconscious effects.

A further mechanism this study investigated was expectation. Does a positive treatment expectation predict the outcome of the OLP response? We observed no relationship of the treatment expectation with the amount of OLP-related changes in emotional distress. Interestingly, we found that the treatment expectation predicted the *perceived* OLP related change, which subjects rated at the end of the experiment, but these perceived changes were not correlated to the actual changes of emotional distress in the OLP group. We observed the same pattern also in our previous study on test anxiety^55^. Hence, the participants do not seem to be aware of how much the OLPs may have worked. In other words, OLP changes seem to work in an unconscious way.

We also tested the role of the belief in OLPs. In contrast to treatment expectation, the belief in OLP predicted the success of the OLP treatment. What is the difference between expectation and belief in OLPs in our study? One can agree to the idea that OLPs may work but may still not believe that they work for him or her in the current trial. This is also supported by a recent qualitative study on OLP effects, in which the patients claimed that the idea that placebos work is plausible, but whether they would work for themselves in the current study was seen as unlikely^15^ (see also our relatively low positive treatment expectations in Table 2). The authors conclude that this may be the reason why asking participants about their positive treatment expectation at the beginning of the study do not predict the OLP responses. In fact, the results of their study^13^ as well as of our own results and other studies (e.g.,^12,16,18^) support this view and do not report that a positive treatment expectation predicts the OLP responses. The authors suggest focusing more on hope rather than positive treatment expectations when addressing OLP mechanisms, especially in patients with chronic diseases^15,22,56^.

However, other studies did find that positive treatment expectations are linked to the OLP response (e.g.,^17^). It remains to be cleared why some studies report a role for treatment expectation while others do not. Relevant factors may be including patient relative to healthy samples, laboratory experimental settings relative to more real life scenarios, or the way how positive treatment expectation is measured^15^.

A further difference between rating belief and expectation in our study are the different time points when these ratings were done. Thus, expectation was assessed at the beginning of the experiment (before receiving information on the power of placebos), whereas we asked participants about the belief at the end of the trial (not only after receiving information on placebos and OLPs, but also after having made the experience receiving OLPs). Thus, we cannot fully exclude that treatment expectations may not have predicted placebo responses because participants lacked information on placebos, or that the OLP belief might be linked to the placebo effect because the individuals felt that the treatment had helped them.

However, the belief in OLP was not related to the perceived success of the OLP treatment (which in turn was not linked to the real effect of the OLPs, as noted above). Hence, since neither the treatment expectation at the beginning of the trial nor the perceived placebo effects were linked to the actual placebo-related change, we speculate that OLP changes may predominantly rely on unconscious processes rather than the conscious expectation of a success. This view is also supported by recent imaging studies that did not find a role for the dorsolateral prefrontal cortex in OLP effects^37,57,58^, which have been described as crucial for (concealed) placebo changes based on expectation^7^.

In the current study we also examined whether the OLP effect is stronger for participants who believed in homeopathic treatments, which are known to be based on placebo effects^59^. On the one hand, homeopathic treatments are very similar to OLP pills. In both treatments the patients know that they receive inert pills without active ingredients (or so few active ingredients that they usually cannot be detected). On the other hand, there might be some differences, since OLP treatments (in contrast to homeopathic treatments) often do not pretend or promise that they *will* help but rather suggest that they *might* help, and patients should give them a try. However, our results do not support the hypothesis that individuals who believe in homeopathic treatments show stronger OLP effects, but interestingly homeopathic belief was linked to positive treatment expectation. Thus, a general (conscious) positive expectation may be linked to belief in homeopathic treatments^60^, but not to OLP related changes. We also found that participants who believed in the power of homeopathic treatments showed higher general sensitivity to stressful stimuli, suggesting a higher “sensitivity” for individuals who are convinced by homeopathic treatments. This higher sensitivity was not found for OLP effects.

This study is not without limitations. Our sample size is not balanced with respect to gender and consists predominantly out of young students. Furthermore, it has to be noted that although the regression model suggested that belief in OLP was a predictor for the placebo response, this model demonstrated only a trend for significance. Therefore, it seems likely that there are also other factors that contribute to this multidimensional behavioral intervention (suggesting several different mechanisms of the OLP effect). Moreover, the current study is a laboratory experiment, with limited transferability to real world stress. Last, several participants, particularly from the OLP group, were lost during the post-measurement for technical reasons. However, we consider it unlikely that this led to systematic bias, as it was a technical error (problem in data storing) that was not systematically related to the experimental conditions.

Taken together, we suggest that rather unconscious than conscious (expectation) processes may be crucial for the success of an OLP treatment. However, the data of the current study do not explain more in detail the underlying mechanisms of these unconscious processes. Further work is needed to explain how these processes may work. This also includes approaches based on the embodiment theory. Although our implantation of a manipulation of those processes did not show an effect on the OLP responses, more studies are needed to also explore the possible role of embodiment in OLP treatments. This will further help to understand the mechanisms of this intriguing multimodal behavioral intervention, which seems to be important when translating OLP results into clinical trials.

## Supplementary Information

Below is the link to the electronic supplementary material.

Supplementary Material 1

## Data Availability

The data are available from the corresponding author upon reasonable request.
