# Supplementary material for: Roles of administration route, expectation, and belief in placebos in a randomized controlled trial with open-label placebos
Source: Sci Rep. 2025 Dec 2;15:42915. doi: 10.1038/s41598-025-27622-5 (PMC12672643; doi:10.1038/s41598-025-27622-5)
Supplement: Supplementary file 1 — Supplementary Material 1 [file 41598_2025_27622_MOESM1_ESM.pdf]

Supplementary Material

Roles of administration route, expectation, and belief in placebos in a randomized controlled trial with open-label placebos

Michael Schaefer*, Carolin Liedtke, Sören Enge

Medical School Berlin, 12247 Berlin, Germany

S1: Questions to examine general belief in placebos

S2: Questions to examine belief in OLP

S3: Question to examine positive treatment expectation

S4: Question to examine attitude towards homeopathic treatments

S1: Questions to examine general belief in placebos

These 4 questions are taken from Leibowitz et al. (2019). Statements had to be rated on a 11-point scale from 0 (definitely not true) to 10 (definitely true):

"Placebo effects are a part of all active medications."
"Placebo effects can occur in all illnesses and conditions."
"Placebo effects happen because the mind has the power to heal."
"Placebo effects work because placebos influence people's expectations about a particular treatment."

S2: Questions to examine belief in OLP

Five items examined the belief in OLPs. They were embedded in other more general belief items on pain. Questions are taken from Guevarra et al., 2020. Participants had to rate the statements on a 11-point scale from 0 (very low) to 10 (very high).

"A placebo can still work on me even though I know that I am taking a placebo."
"In order for placebos to work, the person needs to be deceived into believing they are taking an actual medicine."
"A placebo can reduce my negative emotions even though I know I am taking a placebo."
"A placebo only works if the person is deceived into thinking they are taking an actual medicine."
"A placebo can reduce my pain even though I know that I am taking a placebo."

S3: Question to examine the positive treatment expectation

For the placebo groups we asked the participants to rate the following statement on a visual analogue scale with the ends "not at all" and "very strong".

"In this study, we are attempting to reduce your unpleasant feelings through a special placebo treatment. How likely do you think this placebo treatment is to reduce your negative feelings? Please mark your answer with a cross on the line."

S4: Question to examine attitude towards homeopathic treatments

Participants were asked to rate the following 4 statements on a 11-point scale from 0 (very low) to 10 (very high).

"Do you treat others or yourself with homeopathic treatments?"
"Do you think that homeopathic treatments sometimes may help?"
"Do you think that therapies outside conventional medicine may also be justified?"
"Do you think that not only evidence-based procedures should be considered for medical treatments?"
